# Disparity and Factors Associated With Internet Health Information Seeking Among US Adults Living With Diabetes Mellitus: Cross-sectional Study

**DOI:** 10.2196/32723

**Published:** 2022-05-30

**Authors:** Ransome Eke, Khadiza Tul Jannat, Xin Thomas Yang, Jason M Parton

**Affiliations:** 1 Department of Community Medicine Mercer University School of Medicine Columbus, GA United States; 2 College of Communication and Information Sciences The University of Alabama Tuscaloosa, AL United States; 3 Institute of Data and Analytics The University of Alabama Tuscaloosa, AL United States

**Keywords:** diabetes, internet, information seeking, adults living with diabetes, predictors, racial disparity, Health Information National Trends Survey, race, ethnicity, chronic conditions, self-management, internet health information, mobile phone

## Abstract

**Background:**

Many patients with chronic medical conditions search the internet to obtain medical advice and health information to improve their health condition and quality of life. Diabetes is a common chronic disease that disproportionately affects different race and ethnicity groups in the United States. In the existing literature on the popularity of internet health information seeking among persons with a chronic medical condition, there are limited data on US adults living with diabetes.

**Objective:**

This study aims to examine the factors associated with internet health information seeking among US adults living with diabetes and whether there is a disparity in internet health information seeking stratified by race and ethnicity.

**Methods:**

We conducted a cross-sectional study using the Health Information National Trends Survey data from 2017 to 2020. We selected our study sample based on respondents’ reports on whether they were told they had diabetes, and our primary outcome was internet health information–seeking behavior. We used 2 multivariable logistic regression models to examine the effects of sociodemographic factors and other covariates on the internet health information–seeking behavior of adults with diabetes. Jackknife replicate weights were used to provide bias-corrected variance estimates.

**Results:**

Our study sample included 2903 adults who self-reported that they had diabetes. In total, 60.08% (1744/2903) were non-Hispanic White individuals, 46.88% (1336/2850) were men, and 64% (1812/2831) had some college or graduate education. The prevalence of internet health information seeking in this population was 64.49% (1872/2903), and the main factors associated with internet health information seeking included education level (some college vs less than high school: odds ratio [OR] 1.42, 95% CI 1.44-1.88; and college graduate or higher vs less than high school: OR 2.50, 95% CI 1.79-3.50), age (age group ≥65 years vs age group 18-44 years: OR 0.46, 95% CI 0.34-0.63), and household income level (*P*<.001). In addition, we found significant differences in the effects of predictors stratified by race.

**Conclusions:**

The findings from this study suggest that internet health information seeking is common among US adults living with diabetes. Internet health information could influence the relationship between health care providers and adults living with diabetes and improve their self-management and quality of life.

## Introduction

### Background

Health information seeking through internet platforms is increasingly popular [1-3]. An abundance of research has been conducted to explore why people look for health information, what types of health information they seek, how it influences individuals’ behaviors, and who are more likely to seek health information on web-based platforms [3-7]. Commonly, people search for health information using internet technology to access relevant health information outside a health care facility. In addition, a positive connection between sociodemographic status and the frequency of health information seeking using the internet has been established [8]. For instance, underprivileged groups are more likely to use web-based health information than the majority groups [9], and being younger and female has been found to be a consistent predictor of eHealth use [10]. Other factors linked to the frequency of web-based health information seeking include income, sex, race and ethnicity, age, and the exposure level of an individual [1,11].

Diabetes mellitus is a common chronic medical condition that disproportionately affects the US adult population. Results obtained from the 2011 to 2016 National Health and Nutrition Examination Surveys data indicated that the prevalence of total diabetes among adult non-Hispanic White individuals with diagnosed diabetes was approximately 12%. In non-Hispanic Black and Hispanic individuals, the prevalence was approximately 20% and 22%, respectively [12]. Studies show that diabetes mellitus is a major chronic disease that carries a significant socioeconomic burden, and the prevalence is projected to rise in the future [13-15]. Management of this condition requires high-quality clinical care and self-management to reduce the risk of associated complications and improve quality of life [11]. Behavior modification and self-management are crucial in effectively managing persons living with diabetes.

### Pattern of Health Information Seeking Among Persons With Diabetes

Research suggests that information accessibility is an efficient tool and support necessary to improve chronic medical conditions, including diabetes [16]. Few studies have described the pattern of health information seeking among persons with diabetes. Studies suggest that persons with diabetes have been engaged in passive or active information-seeking activities [17-19]. Passive information-seeking activities involve reading the newspaper and watching television, whereas active information seeking involves mainly using the internet as a source of health information [17,18]. Morgan and Trauth [20] used the Integrated Model of E-Health Use developed by Dutta-Bergman [21] to investigate eHealth information–seeking behavior among persons with diabetes in Greece. The authors [20] found that people with diabetes exhibited different health information–searching behavior because of the intrinsic motivation resulting from access to health care providers or resources.

There is a shortage of data on the internet health information–seeking pattern among adults with diabetes in the United States. Given the growing popularity of internet health information–seeking behavior and the differences in the prevalence of diabetes in the United States, it is vital to understand the factors that predict the use of the internet to seek health information among US adults with diabetes. In addition, with the reported disproportionate racial prevalence of diabetes in the United States, it is essential to investigate whether there is a racial or ethnic disparity in internet health information seeking. Knowing this information is critical for improving diabetes health education and communication, support systems, and quality of life of adults with diabetes in the United States. This study examines the factors associated with internet health information seeking and racial disparity in internet health information seeking among US adults with diabetes.

## Methods

### Data Source

This cross-sectional study uses data from the Health Information National Trends Survey (HINTS). HINTS is a national representative survey that collects data from the US noninstitutionalized adult population [22]. Conducted by the National Cancer Institute, the survey assesses trends in health information seeking, health information technology adoptions, health communication, knowledge, attitudes, and behavior.

To identify our study population, we pooled and combined data from 4 administrations of HINTS: 2017 (version 5, cycle 1, N=3285), 2018 (version 5, cycle 2, N=3504), 2019 (version 5, cycle 3, N=3374), and 2020 (version 5, cycle 4, N=3865). This study focused on investigating internet health information seeking among the adult population with diabetes. We selected respondents who answered “Yes” to the question “Has a doctor or other health professional ever told you that you had diabetes or high blood sugar?” A total of 2903 respondents met the inclusion criteria for this study (655/3285, 19.94%, in 2017; 714/3504, 20.38%, in 2018; 717/3374, 21.25%, in 2019; and 817/3865, 21.14%, in 2020).

### Ethics Approval

This study was approved as exempt by the institutional review board of the University of Alabama because no human participants were involved.

### Dependent Variable

Our dependent variable, internet health information–seeking behavior, was defined on the basis of the respondents’ report on whether they had in the past 12 months used a computer, smartphone, or other electronic means to look for health or medical information for themselves (yes or no). We excluded invalid or missing responses (52/2903, 1.79%) in our final analyses because the percentage was very small.

### Predictor Variables

The primary predictor variables of interest in this study included sociodemographic information: race and ethnicity (non-Hispanic White, non-Hispanic Black, and other), sex (male and female), age group (18-44 years, 45-64 years, and ≥65 years), education level (less than high school, high school graduate, some college, and college graduate or higher), occupation (employed and unemployed), household income (<US $50,000, US $50,000 to <US $75,000, and ≥US $75,000), residency (urban and rural), and marital status (married, divorced, widowed, single, or never been married). Other covariates included were frequency of visits to health care providers (≤1 time, 2-4 times, and ≥5 times), insurance type (private, public, mixed, no insurance, and other), quality of care (excellent or very good, good, and fair or poor), general health (excellent or very good, good or fair, and poor), ability to take care of one’s health (completely or very confident, somewhat confident, and a little or not confident at all). We also examined the respondents’ level of trust in the different sources of information (medical professionals, internet, social network, traditional media, and organizations). The trust scores were reverse coded: 4=a lot, 3=some, 2=a little, and 1=or not at all. Medical professionals as a source of information was scored using only 1 question: “From a doctor?” The social network score was based on the mean of 2 questions: “From family or friends?” and “From religious organizations or leaders?” The internet score was based on 1 question: “Internet?” The traditional media score was based on the mean of 2 questions: “From radio?” and “From television?” The newspapers and magazines score was based on 1 question: “From newspapers or magazines?” The trust in organizations score was based on the mean of 2 questions: “From government health agencies?” and “From charitable organizations?”

### Statistical Analysis

We used descriptive analyses to summarize the frequencies and unweighted and weighted proportions of respondents grouped by sociodemographic characteristics. The weighted proportions were generated using the survey’s weighting variables to generalize the results to the US population. We calculated the trust score using the original survey questions and estimated the mean trust scores for the different sources of information. Multivariable logistic regression models were created to explore the association between the independent variables and health information–seeking behaviors. A total of 2 multiple logistic regression models were constructed to determine the impact of variables of interest with covariates (model 1) and without covariates (model 2). Jackknife replicate weights were used to provide bias-corrected variance estimates [22]. All analyses were conducted using SAS software (version 9.4; SAS Institute Inc), and *P*<.05 was considered statistically significant.

## Results

### Weighted and Unweighted Estimates

**Figure 1:**
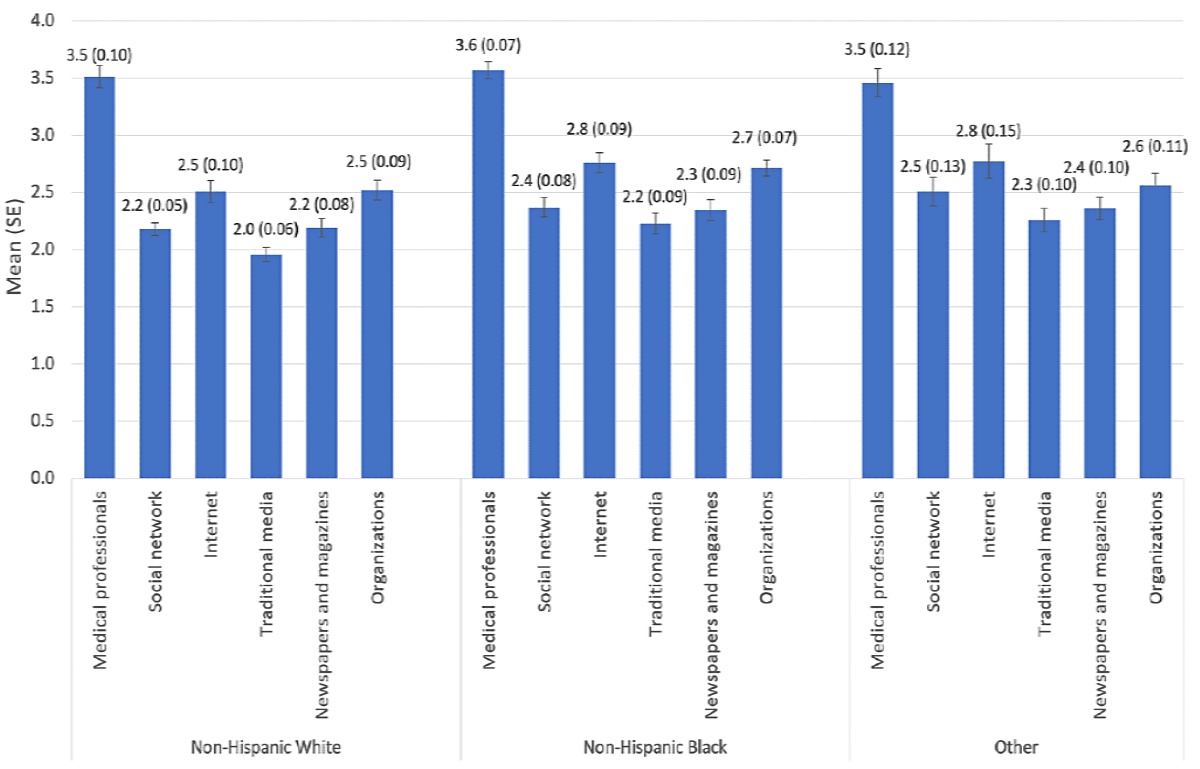
Mean scores of trust in health information sources stratified by race and ethnicity group.

The weighted and unweighted estimates of the characteristics of interest are summarized in Table 1. A total of 2903 respondents with self-reported diabetes were selected. In total, 60.08% (1744/2903) of the respondents were non-Hispanic White individuals, and male respondents accounted for 46.88% (1336/2850) of the total samples. A little more than half of the respondents were aged ≥65 years (1463/2903, 50.4%), and 64% (1812/2831) had some college or graduate education. Most of the respondents lived in urban areas (2519/2903, 86.77%) or reported having an annual household income of <US $50,000 (1460/2568, 56.85%). Figure 1 shows that medical professionals were the most trusted among all sources of health information, meaning the scores were not significantly different across races (*P*=.12). In addition to medical professionals, patients (or people) with diabetes also trust the internet and organizations, and no significant differences were found among the 3 racial groups. The trust in traditional media on health information was lowest in each racial group compared with the trust in other sources. The trust in traditional media was significantly lower in the non-Hispanic White group than in the non-Hispanic Black and other groups (*P*<.001). Trust in social networks, newspapers, and magazines was also not different among the different racial groups (non-Hispanic White, non-Hispanic Black, and other groups). Table 2 presents the characteristics of the respondents who searched the internet for health information compared with those who did not search the internet for health information. Overall, 61.76% (1793/2903) reported that they searched the internet for health information for themselves. Among those who responded yes (1793/2903, 61.76%) to whether they used the internet for health information, most were women (959/1769, 54.21%), non-Hispanic White individuals (1118/1793, 62.35%), residing in an urban area (1595/1793, 88.96%), and married (998/1766, 56.51%). There were significant differences in age group (*P*<.001), education level (*P*<.001), occupation (*P*=.01), sex (*P*=.03), and household income (*P*<.001). In addition, we observed a significant relationship between marital status (*P*<.001) and insurance types of respondents (*P*=.002) and the internet health information seeking among other races with diabetes.

**Table 1:** Sample characteristics of respondents with diabetes, of Health Information National Trends Survey, 2017 to 2020 (N=2903).

Variable			Value, n		Unweighted estimates (%)		Weighted estimates (%)
**Race and ethnicity**							
	Non-Hispanic White	1744		60.08		65.96	
	Non-Hispanic Black	638		22		16.8	
	Other	521		17.9		17.3	
**Sex**							
	Male	1336		46.88		48.91	
	Female	1514		53.12		51.09	
**Age group (years)**							
	18 to 44	306		10.5		17.8	
	45 to 64	1134		39.06		48.96	
	≥65	1463		50.40		33.25	
**Education**							
	Less than high school	337		11.9		13.8	
	High school graduate	682		24.1		28.5	
	Some college	908		32.1		37.8	
	College graduate or higher	904		31.9		19.9	
**Residency**							
	Urban	2519		86.77		84.84	
	Rural	384		13.2		15.2	
**Marital status**							
	Married	1426		50.46		57.38	
	Divorced	575		20.3		12.1	
	Widowed	433		15.3		8.6	
	Single or never been married	392		13.9		21.9	
**Household income (US $)**							
	<50,000	1460		56.85		54.02	
	50,000 to <75,000	429		16.7		17.4	
	≥75,000	679		26.4		28.6	

**Table 2:** Comparison of characteristics between Health Information National Trends Survey (2017 to 2020) respondents who reported that they searched the internet for health information and those who did not search the internet for health information (N=2903).

Variable			Searched the internet for health information, n (%)					*P* value
			Yes (n=1793)		No (n=1110)			
**Age group (years)**								<.001
	18 to 44	226 (12.6)		80 (7.21)				
	45 to 64	798 (44.51)		336 (30.27)				
	≥65	769 (42.89)		694 (62.52)				
**Race and ethnicity**								.41
	Non-Hispanic White	1118 (62.35)		626 (56.4)				
	Non-Hispanic Black	354 (19.74)		284 (25.59)				
	Other	321 (17.9)		200 (18.02)				
**Education**								<.001
	Less than high school	106 (6)		231 (21.73)				
	High school graduate	316 (17.87)		366 (34.43)				
	Some college	616 (34.84)		292 (27.47)				
	College graduate or higher	730 (41.29)		174 (16.37)				
**Occupation**								.01
	Employed	355 (19.8)		104 (9.37)				
	Unemployed	1438 (80.2)		1006 (90.63)				
**Household income (US $)**								<.001
	<50,000	789 (48.02)		671 (72.54)				
	50,000 to <75,000	300 (18.26)		129 (13.95)				
	≥75,000	554 (33.72)		125 (13.51)				
**Sex**								.03
	Male	810 (45.79)		526 (48.66)				
	Female	959 (54.21)		555 (51.34)				
**Residency**								.15
	Urban	1595 (88.96)		924 (83.24)				
	Rural	198 (11.04)		186 (16.76)				
**Marital status**								<.001
	Married	998 (56.51)		428 (40.38)				
	Divorced	334 (18.91)		241 (22.73)				
	Widowed	180 (10.19)		253 (23.87)				
	Single or never been married	254 (14.38)		138 (13.02)				
**Insurance type**								.001
	Private	566 (31.57)		173 (15.59)				
	Public	546 (30.45)		521 (46.94)				
	Mixed	540 (30.12)		333 (30)				
	Other	39 (2.18)		20 (1.8)				
	No insurance	102 (5.69)		63 (5.68)				
**Quality of care**								.60
	Excellent or very good	1224 (73.82)		729 (75.86)				
	Good	331 (19.96)		164 (17.07)				
	Fair or poor	103 (6.21)		68 (7.08)				
**General health**								.29
	Excellent or very good	479 (26.88)		272 (24.66)				
	Good	1215 (68.18)		746 (67.63)				
	Fair or poor	88 (4.94)		85 (7.71)				
**Ability to take care of health**								.05
	Completely or very confident	1071 (59.87)		691 (62.7)				
	Somewhat confident	579 (32.36)		304 (27.59)				
	A little or not confident at all	139 (7.77)		107 (9.71)				

### Factors Associated With the Use of the Internet to Seek Health Information

The 2 models we created to examine the factors that predict the use of the internet to seek health information in participants who reported that they had diabetes are presented in Table 3. In model 1 (the effect of the main predictors without covariates), the results showed that a higher education level was significantly associated with internet health information seeking compared with a less than high school education level (some college: odds ratio [OR] 1.42, 95% CI 1.44-1.88; college graduate or higher: OR 2.50, 95% CI 1.79-3.50). The older age group (≥65 years) was less likely to seek internet health information than the younger age group (18 to 44 years; OR 0.46, 95% CI 0.34-0.63). In addition, income level significantly predicted the use of the internet to seek health information among respondents with self-reported diabetes. Respondents with a household income of ≥US $75,000 had 40% higher odds of searching for health information on the internet than those with a household income of <US $50,000 (OR 1.43, 95% CI 1.03-1.99). Furthermore, we found that respondents who had made ≥5 visits to health care providers during the past 12 months were significantly more likely to use the internet to seek health information than those with fewer visits (OR 1.52, 95% CI 1.11-2.10).

Model 2 presents the effect of the main predictor variables of interest without the covariates. In this model, only education level, age group, and household income level remained the main predictors of use of the internet to search for health information among persons who reported that they had diabetes. In models 1 and 2, race and ethnicity, occupation, sex, marital status, and urbanity were not significantly associated with using the internet to search for health information among respondents who have diabetes (*P*>.05).

Table 4 shows the multivariable analyses of our main predictor variables and covariates on internet health information seeking stratified by race and ethnicity groups (non-Hispanic White, non-Hispanic Black, and other races). We observed significant differences among the several factors across different race and ethnicity groups. In the non-Hispanic White group, the respondents who seek health information on the internet were more likely to have college graduate or higher degrees, ≥5 visits to health care providers, and no insurance, although older age was significantly associated with lower odds of seeking health information on the internet (OR 0.53, 95% CI 0.34-0.82). Among the non-Hispanic Black respondents, individuals who use the internet to seek health information were more likely to have a household income of US $75,000, whereas those living in a rural area (OR 0.40, 95% CI 0.25-0.66) and the older age group (OR 0.32, 95% CI 0.14-0.72) were associated with lower odds of using the internet to seek health information. Among respondents who were neither non-Hispanic White nor non-Hispanic Black in terms of race and ethnicity, those seeking health information on the internet were more likely to have some college degree, have a household income between US $50,000 and US $75,000, live in an urban area, and be widowed.

**Table 3:** Odds ratios (ORs) and 95% CIs of respondents seeking health information by multiple logistic regression model.

Variable			Model 1, OR (95% CI)		Model 2, OR (95% CI)
**Education**					
	Less than high school (reference)	—^a^		—	
	High school graduate	0.82 (0.61-1.10)		0.82 (0.60-1.10)	
	Some college	1.44 (1.10-1.88)^b^		1.45 (1.12-1.86)^b^	
	College graduate or higher	2.50 (1.79-3.50)^b^		2.76 (2.04-3.74)^b^	
**Occupation**					
	Employed (reference)	—		—	
	Unemployed	0.99 (0.73-1.33)		0.98 (0.79-1.23)	
**Age group (years)**					
	18 to 44 (reference)	—		—	
	45 to 64	0.88 (0.63-1.22)		0.94 (0.72-1.22)	
	≥65	0.46 (0.34-0.63)^b^		0.46 (0.35-0.60)^b^	
**Household income (US $)**					
	<50,000 (reference)	—		—	
	50,000 to <75,000	0.82 (0.63-1.08)		0.88 (0.69-1.11)	
	≥75,000	1.43 (1.03-1.99)^b^		1.41 (1.07-1.87)^b^	
**Residency**					
	Urban (reference)	—		—	
	Rural	0.77 (0.59-1.00)		0.81 (0.65-1.02)	
**Marital status**					
	Married (reference)	—		—	
	Divorced	0.85 (0.61-1.19)		0.87 (0.66-1.15)	
	Widowed	0.85 (0.59-1.22)		0.89 (0.62-1.27)	
	Single, never been married	0.92 (0.58-1.44)		0.87 (0.58-1.30)	
**Sex**					
	Male (reference)	—		—	
	Female	1.12 (0.94-1.34)		1.15 (0.99-1.34)	
**Race and ethnicity**					
	Non-Hispanic White (reference)	—		—	
	Non-Hispanic Black	1.12 (0.79-1.59)		1.05 (0.80-1.39)	
	Other	0.95 (0.61-1.46)		0.92 (0.67-1.26)	
**Insurance type**					
	Private (reference)	—		N/A^c^	
	Public	0.88 (0.54-1.45)		N/A	
	Mixed	1.25 (0.79-2.00)		N/A	
	Other	0.54 (0.15-1.98)		N/A	
	No insurance	1.40 (0.55-3.54)		N/A	
**Frequency of visits to health care providers**					
	≤1 time (reference)	—		N/A	
	2 to 4 times	0.88 (0.63-1.22)		N/A	
	≥5 times	1.52 (1.11-2.10)^b^		N/A	
**Quality of care**					
	Excellent or very good (reference)	—		N/A	
	Good	1.16 (0.80-1.69)		N/A	
	Fair or poor	1.02 (0.68-1.52)		N/A	
**General health**					
	Excellent or very good (reference)	—		N/A	
	Good or fair	1.06 (0.82-1.37)		N/A	
	Poor	0.70 (0.43-1.14)		N/A	
**Ability to take care of health**					
	Completely or very confident (reference)	—		N/A	
	Somewhat confident	1.21 (0.89-1.64)		N/A	
	A little or not confident at all	0.97 (0.62-1.52)		N/A	

^a^Reference level for corresponding predictors.

^b^*P* values met the threshold for statistical significance.

^C^N/A: not applicable (variables were included in model 1 only).

**Table 4:** Odds ratios (ORs) and 95% CIs of respondents seeking health information on the internet by race and ethnicity group.

Variable		Model 1: non-Hispanic White, OR (95% CI)	Model 2: non-Hispanic Black, OR (95% CI)	Model 3: other, OR (95% CI)
**Education**				
	Less than high school (reference)	—^a^	—	—
	High school graduate	0.70 (0.49-1.01)	1.23 (0.58-2.61)	0.57 (0.20-1.58)
	Some college	1.31 (0.95-1.79)	1.36 (0.76-2.45)	4.73 (2.23-10.01)^b^
	College graduate or higher	2.77 (1.93-3.96)^b^	1.72 (0.73-4.03)	2.10 (0.53-8.26)
**Occupation**				
	Employed (reference)	—	—	—
	Unemployed	0.97 (0.69-1.36)	0.90 (0.50-1.63)	0.70 (0.35-1.41)
**Age group (years)**				
	18 to 44 (reference)	—	—	—
	45 to 64	0.81 (0.52-1.26)	1.05 (0.46-2.39)	1.33 (0.49-3.59)
	≥65	0.53 (0.34-0.82)^b^	0.32 (0.14-0.72)^b^	0.30 (0.09-1.04)
**Household income (US $)**				
	<50,000 (reference)	—	—	—
	50,000 to <75,000	0.93 (0.64-1.36)	0.74 (0.36-1.53)	0.35 (0.18-0.70)^b^
	≥75,000	1.47 (0.97-2.24)	2.42 (1.09-5.38)^b^	1.37 (0.74-2.53)
**Sex**				
	Male (reference)	—	—	—
	Female	1.06 (0.85-1.33)	1.18 (0.74-1.88)	1.95 (0.90-4.22)
**Frequency of visits to health care providers**				
	≤1 time (reference)	—	—	—
	2 to 4 times	1.03 (0.67-1.56)	0.62 (0.33-1.17)	0.85 (0.30-2.37)
	≥5 times	1.87 (1.25-2.80)^b^	0.84 (0.42-1.66)	1.48 (0.46-4.74)
**Insurance type**				
	Private (reference)	—	—	—
	Public	0.71 (0.37-1.37)	0.69 (0.36-1.33)	2.76 (0.50-15.09)
	Mixed	1.00 (0.53-1.89)	1.77 (0.68-4.59)	1.24 (0.29-5.36)
	Other	0.53 (0.12-2.43)	1.63 (0.27-9.88)	0.06 (0.01-0.52)^b^
	No insurance	2.63 (1.19-5.81)^b^	0.42 (0.07-2.57)	2.67 (0.52-13.69)
**Residency**				
	Urban (reference)	—	—	—
	Rural	0.91 (0.68-1.20)	0.40 (0.25-0.66)^b^	0.22 (0.06-0.77)^b^
**Marital status**				
	Married (reference)	—	—	—
	Divorced	1.02 (0.70-1.47)	0.69 (0.38-1.24)	1.25 (0.38-4.14)
	Widowed	0.93 (0.58-1.49)	1.28 (0.66-2.45)	0.22 (0.07-0.70)^b^
	Single or never been married	0.76 (0.45-1.29)	1.24 (0.62-2.48)	0.68 (0.21-2.24)
**Quality of care**				
	Excellent or very good (reference)	—	—	—
	Good	1.11 (0.70-1.74)	1.64 (0.75-3.58)	0.70 (0.23-2.16)
	Fair or poor	1.27 (0.69-2.32)	0.58 (0.25-1.35)	0.90 (0.18-4.43)
**General health**				
	Excellent or very good (reference)	—	—	—
	Good	0.92 (0.68-1.24)	1.22 (0.63-2.36)	1.51 (0.54-4.27)
	Fair or poor	0.71 (0.40-1.26)	1.35 (0.35-5.13)	0.29 (0.05-1.82)
**Ability to take care of health**				
	Completely or very confident (reference)	—	—	—
	Somewhat confident	1.27 (0.86-1.87)	1.46 (0.69-3.09)	1.10 (0.41-2.93)
	A little or not confident at all	0.82 (0.50-1.36)	0.76 (0.21-2.71)	3.16 (0.52-19.41)

^a^Reference level for corresponding predictors.

^b^*P*<.05.

## Discussion

### Principal Findings

Diabetes self-management skills refer to the tasks the patient must carry out to manage or reduce the impact of diabetes on their health status and daily living. The internet is a popular platform where individuals with chronic medical conditions obtain information or opinions to improve their health conditions. This cross-sectional study examined the factors associated with internet health information–seeking behavior among US adults with diabetes. We found that approximately two-thirds of the individuals who reported that they are living with diabetes seek personal health information using the internet. Standard features of the US adults with diabetes who seek internet health information include non-Hispanic White race, some college or graduate-level education, unemployment, being married, women, and living in urban areas. The significant predictors of internet use for health information are education level, age, household income, and frequency of visits to health care provider. Our results show that persons with college graduate–level education or higher have 2.5 times higher odds of seeking health information from the internet than individuals with less than high school education. People with diabetes who frequently visit health care providers (≥5 times per year) are 1.5 times more likely to seek health information from the internet than those who see their provider once or not at all in a year. Older age groups (≥65 years) are significantly less likely to use the internet for health information than younger age groups. We observed inconsistencies, by race, in the factors associated with internet health information seeking among US adults with diabetes. The main predictors of internet health information seeking among non-Hispanic White individuals are college graduate education or higher degree, younger age, no insurance, and higher frequency of visits to health care providers. By contrast, among non-Hispanic Black individuals, the main predictors are higher household income, residency, and age of patients.

The ever-growing availability of the internet increases its utility for accessing health information, especially among people with chronic medical conditions. Even so, health care professionals remain the most trusted source of health information and are trailed by internet sources. As in most studies, we observed that the trust in health information sources among US adults with diabetes was higher for health care professionals than for internet sources [19,23,24]. Even so, our study shows that a large proportion of US adults living with diabetes seek health information using internet sources. This finding supports the high rate of reported internet health information–seeking behavior among persons with chronic medical conditions. Data show that >50% of the adults living with chronic medical conditions have accessed the internet for health information related to their situation. Furthermore, 36% reported that information obtained from the internet was helpful regarding medical advice and health information [25].

Contrary to our findings of a large proportion of US adults with diabetes seeking internet health information, Kalanzi et al [24], in their study conducted in Greece, observed a low ranking in the utility of the internet among their study participants. The differential health information–seeking behavior observed between these 2 populations could be explained by their intrinsic motivations, such as access to health care providers and available resources. Nevertheless, our study suggests that people living with diabetes are becoming better informed and better understand their health problems because of their increasing internet health information–seeking behavior. In addition, this study provides essential information to improve the relationship between health care providers and persons living with diabetes. Establishing a good relationship will improve the management and quality of life of individuals living with diabetes. It is vital for health care providers to actively engage with persons living with diabetes in the decision-making process when caring for them. In addition, health care providers should consider discussing available internet-based resources in their management plan to enhance the use of appropriate resources and accuracy of diabetes health information obtained from the internet source.

Individual characteristics (eg, income, sex, race and ethnicity, age, and education) influence internet health information–seeking behaviors, regardless of the types of illnesses [9,26-28]. There is a positive relationship between individuals with chronic diseases and the frequency of internet health information seeking, which influences their health behavior changes [29]. Our study found that age, education, and household income were significant factors influencing internet health information seeking among adults living with diabetes mellitus. This finding supports the existing literature [30]. Trust in the source of information influences the connection between age and internet health information seeking. For instance, older people, compared with the young generation, find their physicians or health care providers to be reliable sources for seeking health information compared with internet use [31]. In addition, technology adaptation and trust intersect regarding internet health information seeking between young and older adults. Compared with older adults, younger adults are more likely to adopt the internet to search for health information and to trust health information found on the internet because technology adaptation enables them to differentiate between websites that contain low-quality health information and those that contain high-quality health information [31]. These findings emphasize the connection between technology adaptation or acceptance and eHealth literacy regarding health information seeking [31]. Furthermore, levels of education make a huge difference in internet health information seeking, as described in several studies [8,10]. For instance, individuals with higher levels of education are more likely to seek internet health information than those with lower education. We observed 2.5 times the odds of internet health information seeking in adults with diabetes and some college education or higher degree compared with those with less than high school education. This observation underscores the significance of the role of social determinants in promoting health and health equity for all [32] because “social determinants of health are the conditions in which people are born, grow, live, work and age that shape health” [33].

Overall, our study showed a significant association between higher internet use for health information and higher education levels in all race categories. We did not find any significant association between the race of a person living with diabetes and internet health information–seeking behavior. However, we observed inconsistency in the predictors of internet health information seeking across racial groups of adults living with diabetes. Although no association was observed between insurance types and internet use among the Hispanic and non-Hispanic Black individuals with diabetes, our results show that non-Hispanic White individuals with diabetes who have no insurance are significantly more likely to use the internet for health information than non-Hispanic White individuals with private insurance. Previous reports of the association between internet use and insurance status are mixed. Research mostly shows that people with private insurance are more likely to use the internet to seek health information, which could be attributed to their socioeconomic status [34-36]. However, our findings support a previous report that uninsured persons with a reported chronic medical condition were more likely than those with private insurance to search the internet for health information [37]. This finding could imply that higher internet health information–seeking behavior among persons without health insurance and who have a chronic medical condition such as diabetes may be due to barriers in accessing health services because of their insurance status. Research also shows that individuals who have easy access to health information through their health care providers are less likely to search the internet for health information because they have better access to health care services [38].

In comparison, among non-Hispanic Black individuals with diabetes, the main significant predictors of internet health information seeking include higher household income and living in an urban area. Notably, our study explored the difference in the effects of the predictor on internet health information seeking stratified by race and ethnicity among US adults with diabetes. We were unable to compare our data with any similar studies. However, studies have shown a vast racial divide in internet health information–seeking behavior [29,39-41]. These studies indicate that non-Hispanic Black individuals seek more internet health information than non-Hispanic Black and other races to obtain personal health information and medical advice. For instance, Lorence et al [39], in their study, observed a significant gap in the access to the internet between non-Hispanic White and minority races, with the non-Hispanic White group having more access to the internet for health information than the non-Hispanic Black and Hispanic groups [39]. Further research is needed to explore further the coeffect of race and predictors of internet health information–seeking behavior among US adults with diabetes.

The findings from our study add significantly to the literature; however, the study is not without limitations. First, the data used in this study, HINTS data, are self-report secondary survey data. Therefore, there may be issues with validity and bias in the information collected in this survey. For example, the identification of persons with diabetes is based on the information provided by the respondents. We could not verify this information by using clinical data to determine whether diabetes was diagnosed clinically in these respondents. In addition, the response to our dependent variable could have been overreported or underreported. Second, the HINTS data are cross-sectional data. We could not ascertain the trend in internet information seeking in this population and examine any behavior change during the study period. Third, our analytical approach may be subject to robustness issues related to sample sizes. The small sample size of non-Hispanic Black and other race strata compared with the non-Hispanic White group could have affected our findings in this study. Our pooled approach and use of jackknife weights in our analyses helped minimize potential sampling biases and enhance the generalizability of our results. Even with these limitations, the nationwide sampling approach of the survey data is a great strength of this study.

### Conclusions

Our study provides insights into the predictors of internet health information–seeking behavior of US adults living with diabetes. Seeking internet health information is common among adults living with diabetes. To improve the self-management and quality of life of individuals living with diabetes, it is crucial for health care providers to educate patients about reliable and verifiable internet health information sources.

## Abbreviations

HINTS: Health Information National Trends Survey
OR: odds ratio
